# Occurrence of simple sequence repeats in cDNA sequences of safflower (*Carthamus tinctorius*) reveals the importance of SSR-containing genes for cell biology and dynamic response to environmental cues

**DOI:** 10.3389/fpls.2022.991107

**Published:** 2022-11-17

**Authors:** Ahmad Jawid Ahmadi, Assadollah Ahmadikhah

**Affiliations:** ^1^ Agronomy Department, Faculty of Agriculture, Higher Education Institute of Samangan, Samangan, Afghanistan; ^2^ Department of Cell and Molecular Biology, Faculty of Life Sciences and Biotechnology, Shahid Beheshti University, Tehran, Iran

**Keywords:** blast, genic SSR, genome-based breeding, GO enrichment, *in silico*

## Abstract

Safflower (*Carthamus tinctorius*) is a diploid crop plant belonging to the family *Asteraceae* and is well known as one of important oilseed crops due to edible oil containing unsaturated fatty acids. In recent years it is gaining increased attention for food, pharmaceutical and industrial uses, and hence the updating its breeding methods is necessary. Genic simple sequence repeats (SSRs) in addition of being desire molecular markers, are supposed to influence gene function and the respective phenotype. This study aimed to identify SSRs in cDNA sequences and further analysis of the functional features of the SSR-containing genes to elucidate their role in biological and cellular processes. We identified 1,841 SSR regions in 1,667 cDNA sequences. Among all types of repeats, trinucleotide repeats were the most abundant (35.7%), followed by hexanucleotide (29.6%) and dinucleotide repeats (22.0%). Thirty five SSR primer pairs were validated by PCR reaction, detected a high rate of polymorphism (>57%) among safflower accessions, physically mapped on safflower genome and could clearly discriminate the cultivated accessions from wild relatives. The cDNA-derived SSR markers are suitable for evaluation of genetic diversity, linkage and association mapping studies and genome-based breeding programmes. Occurrence of SSR repeats in biologically-important classes of proteins such as kinases, transferases and transcription factors was inferred from functional analyses, which along with variability of their repeat copies, can endow the cell and whole organism the flexibility of facing with continuously changing environment, and indicate a structure-based evolution mechanism of the genome which acts as an up-to-dating tool for the cell and whole origanism, which is realized in GO terms such as involvement of most SSR-containing genes in biological, cellular and metabolic processes, especially in response to stimulus, response to stress, interaction to other organisms and defense responses.

## Introduction

Safflower (*Carthamus tinctorius*, 2n=2x=24) is ordinarily related to tribe (*Cynareae*), subfamily (*Tubulifloreae*), and a member of the *Asteraceae* family. The east of the Mediterranean region has known as an origin center for the genus (Ashri and Knowles, 1960). Vavilov (1951) proposed three centers of safflower’s origin including India, Afghanistan, and Ethiopia; whereas Knowles (1969) defined seven origin center for safflower including Far East, India and Pakistan, the Middle East, Egypt, Sudan, Ethiopia and the last one in Europe (Knowles, 1969). The size of haploid genome of the genus ‘*Carthamus’* ranges from 1.36 GB (*C. gypsicola*) to 1.41 GB (*C. palaestinus*) (Garnatje et al., 2006). Cultivated safflower (*C. tinctorius*) is being widely grown across the world and globally is considered as an important crop because of its multi-purpose applications including edible oil products, red dye, and medicinal usages and the use in livestock feeding (Wu et al., 2021). In the mid last century, its recognition as a qualified oil source containing high-unsaturated fatty acid in comparison to other oil crops and industrial usages caused it to be widely cultivated in Asia, Europe, Australia and North America (Knowles, 1955; Weiss, 1971). It is especially popular for its high linoleic acid (LA) and flavonoid contents (Wu et al., 2021). Its two unsaturated fatty acids (linoleic acid (LA) and oleic acid (OA)) are essential for human health; for example it was reported that LA decreases serum cholesterol and risk of cardiovascular events (Ramsden et al., 2016; Marklund et al., 2019). On the other hand, the olive oil which is rich in OA, has a beneficial effect on cancer, autoimmune and inflammatory diseases, and also facilitates wound healing (Sales-Campos et al., 2013).

Different types of molecular markers (mostly arbitrary) have been used to reveal genetic diversity in safflower by several investigators; For example, random amplified polymorphic DNA (RAPD) markers (Sehgal and Raina, 2005; Amini et al., 2008; Khan et al., 2009; Rehman et al., 2015; Talebi and Abhari, 2016), inter simple sequence repeat (ISSR) markers (Sabzalian et al., 2009; Bagmohammadi et al., 2012; Majidi and Zadhoush, 2014), amplified fragment length polymorphism (AFLP) markers (Sehgal and Raina, 2005; Johnson et al., 2007), retrotransposon markers (Ali et al., 2019) and single nucleotide polymorphism (SNP) markers (Zhao et al., 2021). Likewise, Mokhtari et al. (2013) and Golkar and Mokhtari (2018) used the sequence-related amplified polymorphism (SRAP) and start codon targeted (SCoT) markers to determine genetic variation and similarity among safflower species. Moreover, simple sequence repeat (SSR) markers have also been applied in safflower by other researchers for different purposes. For example, Kiran et al. (2017) used 44 SSR markers for genetic diversity evaluation, linkage disequilibrium and population structure analysis among 148 accessions of safflower. Moreover, assessment of genetic distinctiveness among Indian and Mexican cultivars of safflower (Kadirvel et al., 2017), and association mapping of agronomic traits (Ambreen et al., 2018) were reported.

Recently, genic-SSR or expressed sequence tag (EST)-SSR markers were used for investigating the genetic diversity of different crop plants. Developing EST-SSRs through data mining acquired from publicly available databases has made them fast, efficient and moderately inexpensive markers compared to the genomic-SSR markers, as well as their transferability across species (Gupta et al., 2003; Chapman et al., 2009; Singh et al., 2021). The microsatellites existing in the genic regions have a direct role in the genome organization, recombination, gene regulation, quantitative genetic variation, and evolution of genes (Hao, 2018). Gene-based SSRs are sometimes assigned putative function and hence also termed as functionally relevant SSRs (Mishra et al., 2021). Therefore, numerous researches attempted to develop and utilize genic-SSRs in multiple crops. For instance, the genic-SSRs were developed and reported for sugarcane (Cordeiro et al., 2001), barley, maize, rice, sorghum and wheat (Kantety et al., 2002; Gupta et al., 2003; Yu et al., 2004; Peng and Lapitan, 2005; Fu et al., 2006; Varshney et al., 2006), cotton (Park et al., 2005; Han et al., 2006), coffee (Aggarwal et al., 2007), citrus (Chen et al., 2006); barrel medic (Gupta and Prasad, 2009), sorghum (Srinivas et al., 2009), pigeonpea (Dutta et al., 2011), finger millet (Arya et al., 2013; Pandian et al., 2018), mungbean (Gupta et al., 2014), Japanese larch (Chen et al., 2015), mulberry (Thumilan et al., 2016), lettuce (Oliya et al., 2018). In a few investigations the genic-SSRs were reported for oil crops such as sunflower (Pashley et al., 2006), soybean (Hisano et al., 2007), cultivated groundnut (Mondal et al., 2012; Zhang et al., 2012) and sesame (Zhang et al., 2012). Nevertheless, a limited number of studies investigated EST-SSRs in safflower. For instance, more recently Singh et al. (2021) identified EST-based SSRs and used them for assessment of genetic diversity and cross-species transferability in safflower. The use of such markers may be due to their proper characters such as hyper-variability, multi-allelic nature, co-dominant inheritance, reproducibility, relative abundance, intensive genome coverage, chromosome-specific location, feasibility and high-throughput genotyping (Parida et al., 2009). Chapman et al. (2009) generated polymorphic EST-SSRs in safflower and assessed their cross-taxon utility across the genus and the family. EST-SSRs have also used for genetic purity analysis of safflower hybrids, as well (Naresh et al., 2009). Whereas, limited studies were available for the development of genic-SSR markers from safflower cDNA sequences in which the complete length of coding sequences of genes must be screened for presence of SSRs. The microsatellites located in coding regions of genes may impact the coded protein activity and thus can affect the expression of proteins, while their presence in non-coding regions (5’-UTR, 3’-UTR and introns) may be involved in gene regulation and transcription (Lawson and Zhang, 2006). According to the above, the objective of the present study was to develop novel cDNA-SSRs from safflower’s cDNA sequences. Furthermore, we also aimed to characterize the genes containing these tandem repeats and their functions by molecular analyses.

## Materials and methods

### SSR detection

Initially, the full length cDNA sequences data set (39,718 entries) of safflower (*Carthamus tinctorius*) was downloaded from national center for biotechnology information (NCBI) website (www.ncbi.nlm.nih.gov). Subsequently, for detecting SSRs ≥18 nucleotides in length within the subject sequences, the R Biostrings package (matchPDict function) was used (the R scripts are presented in **Supplementary File S1**). Mononucleotide and all possible forms of dinucleotide (NN), trinucleotide (NNN), tetranucleotide (NNNN), pentanucleotide (NNNNN) and hexanucleotide (NNNNNN) repeats were extracted and stored in.txt format for further analyses.

### Sequence characterization

The FGENESH (http://www.softberry.com/berry.phtml?topic=fgenesh&group=programs&subgroup=gfind) or GENESCAN (http://hollywood.mit.edu/GENSCAN.html) software were used to predict existence of genes and putative coding open reading frames (ORFs) in cDNA sequences. The search options in both software were based on Arabidopsis code table, and the rest of the options were all set to default. The basic local alignment search tool for proteins (BLASTp) (protein BLAST) (https://blast.ncbi.nlm.nih.gov/Blast.cgi) was used for identification of putative homologs of genes in protein databases. Consequently, the BLASTp hits with at least 65% coverage and 70% identity were chosen for further analysis.

gProfiler online tool (http://biit.cs.ut.ee/gprofiler/gost/) was used for gene ontology (GO) enrichment. For this purpose, *Arabidopsis thaliana* was selected as a model organism. The GO analysis for genes was done according to molecular function (MF), cellular component (CC), biological process (BP) and Kyoto Encyclopedia of Genes and Genomes (KEGG) pathway. An adjusted p value of <0.05 was considered for denoting significance. Analysis of promoter regions of the given genes was conducted using PlantPAN (http://plantpan.itps.ncku.edu.tw/). The STRING database (https://string-db.org/) was used for interaction analysis of proteins.

### SSR primers, PCR assay and physical mapping

For validation of the identified SSR sequences and their use in subsequent analyses, 38 primer pairs were designed based on the flanking regions of SSR motifs (**Supplemental Table S1**). Where the SSR motif lied in a marginal boundary of cDNA sequence, the cDNA sequence was aligned to newly published genome sequence of safflower (Wu et al., 2021) (https://safflower.scuec.edu.cn/) to obtain the flanking SSR sequences to be used for primer design. Total DNA was extracted by cetyltrimethylammonium bromide (CTAB) method (Dellaporta et al., 1983) from seedlings of 11 safflower accessions including 9 cultivated accessions (*Carthamus tinctorius*) and 2 wild relatives (*C. lanatus* collected from Tehran Province, and *C. oxyacanthus* collected from Golestan Province). Polymerase chain reaction (PCR) reactions were done in 0.2 mL micro tubes. PCR mixture included 5 µL of deionized water, 6 µL PCR Master Mix (CinaClone Co.), 0.25 µL of each primer (10 pg) and 0.75 µL of DNA template (15 ng). The PCR temperature profile was as following: amplification started at 94°C for 5 min and continued for 35 cycles (each cycle consisted of denaturation at 94°C for 35 s, annealing at 55°C for 30 s, extension at 72°C for 1 min) and the extension was done at 72°C for 7 min. The PCR products were separated by electrophoresis in 3% agarose gels and 1x TBE buffer containing 0.5 µg mL^−1^ of ethidium bromide. DNA bands became visible after staining by ethidium bromide and their photos were taken under ultraviolet light by a Gel-Doc system (BioRad, USA).

The physical location of SSR sequences was defined by searching the primer pairs and internal sequences against newly published safflower genome (Wu et al., 2021) (https://safflower.scuec.edu.cn/) using R Biostrings package, matchProbePair and matchLRPatterns functions. The map was drawn using MapChart v.2 (Voorrips, 2002).

## Results

### Identified cDNA-SSRs

**Figure 1:**
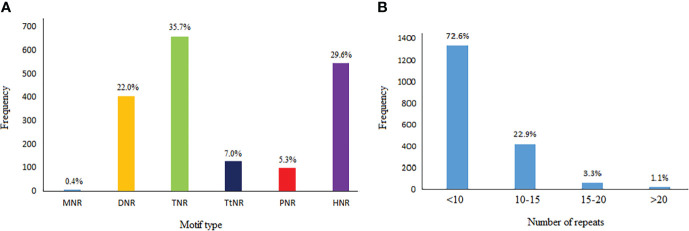
**(A)** Frequency distribution of 6 SSR motif types (viz. mono- to hexanucleotide repeats) in cDNA sequences of safflower. **(B)** The number of repeats vs. frequency of different motifs in identified SSR sequences. MNR, mononucleotide repeat; DNR, dinucleotide repeat; TNR, trinucleotide repeat; TtNR, tetranucleotide repeat; PNR, pentanucleotide repeat; HNR, hexanucleotide repeat.

In 39,817 available safflower cDNA clones, 1,841 (4.62%) microsatellite repeats with SSR length of ≥18 n were detected (**Supplemental Table S2**). Frequency of the extracted repeats based on their types in the safflower cDNA sequences was different: 7 (0.38%) mononucleotides, 405 (22.0%) dinucleotides, 658 (35.74%) trinucleotides, 128 (6.95%) tetranucleotides, 98 (5.32%) pentanucleotides, and 545 (29.6%) hexanucleotides (**Figure 1A**). Among the cDNA sequences, the repeat numbers of SSRs varied from 3 to 28, but repeat numbers of 3-9 were predominant in the SSRs (72.6% of the total SSRs). Repeat numbers of 10-15 accounted for 22.9% of SSRs, repeat numbers of 15-20 accounted for 3.3% of SSRs and repeat numbers more than 20 just were accounted for 1.1% of the SSRs (**Figure 1B**).

Also we found 1, 4, 18, 20, 32 and 115 motif types of mono-, di-, tri-, tetra-, penta- and hexanucleotides in cDNAs of safflower (**Supplemental Table S2**). Longest SSR regions consisted of hexanucleotide (TCCATC)_n_ (with 16 and 18 repeats) which were found in cDNA clonens EL382716.1 and EL381184.1, respectively. Next, AG and TC with 28 repeats were longest dinucleotide SSRs (in EL391333.1 and EL383067.1 clones, respectively). The more abundant motifs within each repeat type were also different. For example, as shown in **Table 1**, among all of 4 possible mononucleotide repeat types [(A)_n_, (T)_n_, (C)_n_ and (G)_n_], only (T)_n_ was found in safflower cDNAs, while in the case of dinucleotide SSRs, (TC)_n_ and (AG)_n_ motifs were prevalent. The motifs such as (TTC)_n_, (ATC)_n_ and (AAG)_n_ in trinucleotide SSRs, (TTTG)_n_ and (ATTC)_n_ in tetranucleotide SSRs, (TTCTC)_n_ and (AATTC)_n_ in pentanucleotide SSRs, and lastly, the (TCCATC)_n_ and (GATGGA)_n_ motifs in hexanucleotide SSRs were more abundant repeats among all expected motifs for the repeat types (see **Supplemental Table S2**).

**Table 1:** Number and frequency of different types of SSR motifs in safflower cDNA sequences (SSR length≥18 bp).

Repeat type (total number)	Motif	Frequency	%
Mononucleotide (7)	(T)n	7	100.0
	Other	0	0.0
Dinucleotide (405)	(TC)n	228	56.3
	(AG)n	83	20.5
	(AC)n	66	16.3
	Other	28	6.9
Trinucleotide (658)	(TTC)n,	212	32.2
	(ATC)n	127	19.3
	(AAG)n	108	16.4
	Other		42.1
Tetranucleotide (128)	(TTTG)n	56	43.8
	(ATTC)n	16	12.5
	Other	56	43.7
Pentanucleotide (98)	(TTCTC)n	18	18.4
	(AATTC)n	14	14.3
	Other	66	77.3
Hexanucleotide (545)	(TCCATC)n	32	5.9
	GATGGA	27	5.0
	(AACCCT)n	25	4.6
	Other	461	84.5

Total number of each repeat type was written in parenthesis.

In total, 1,841 SSR regions were found in 1,667 cDNA clones (e.g. some cDNA sequences harbored more than one SSR region). The distribution of SSR regions is shown in **Table 2**. All mono-, tri-, tetra- and pentanucleotide SSRs (in total 1,434) were perfect (SSR repeat length ≥20 n), and only a small portion of dinucleotide SSRs (4 out of 405, <1%) were imperfect (SSR repeat length <20 n), in contrast most of hexanucleotide SSRs (403/545, ~74%) were imperfect (**Table 3**). All cDNAs which contained mono, tetra- and pentanucleotide motifs (in total 233) carried just one SSR region and in contrast, the cDNAs containing di-, tri- and hexanucleotide motifs carried one or more SSR regions (**Table 3**).

**Table 2:** Distribution of SSR repeats in cDNA clones.

SSR repeat	Total No.	No of cDNAs harboring SSRs	% cDNAs
Mononucleotide	7	7	100
Dinucleotide	405	396	97.8
Trinucleotide	658	635	96.5
Tetranucleotide	128	114	89.1
Pentanucleotide	98	93	94.9
Hexanucleotide	545	422	77.4
Sum	1,841	1,667	

**Table 3:** Distribution of perfect and imperfect SSRs, and cDNAs with 1 or more SSR regions in different types of repeated motifs.

SSR repeat	Imperfect	Perfect	cDNAs with 1 SSR region	cDNAs with >1 SSR region
Mononucleotide	0	7	7	0
Dinucleotide	4	401	395	10
Trinucleotide	0	658	648	10
Tetranucleotide	0	128	128	0
Pentanucleotide	0	98	98	0
Hexanucleotide	403	142	464	81
Sum	407	1434	1740	101

### Results of BLAST searches

1,721 out of 1,841 SSR-containing cDNA sequences, could be identified as protein-coding sequences by ORF prediction tools. Peptide sequences obtained by the software were used as input for BLASTp searches. From these SSR-containing protein-coding sequences, 1,613 sequences showed significant hits with existing genes in database sequences, and after removing duplicates, 713 genes with unique gene IDs were selected for subsequent analyses (**Supplemental Table S3**).

**Figure 2:**
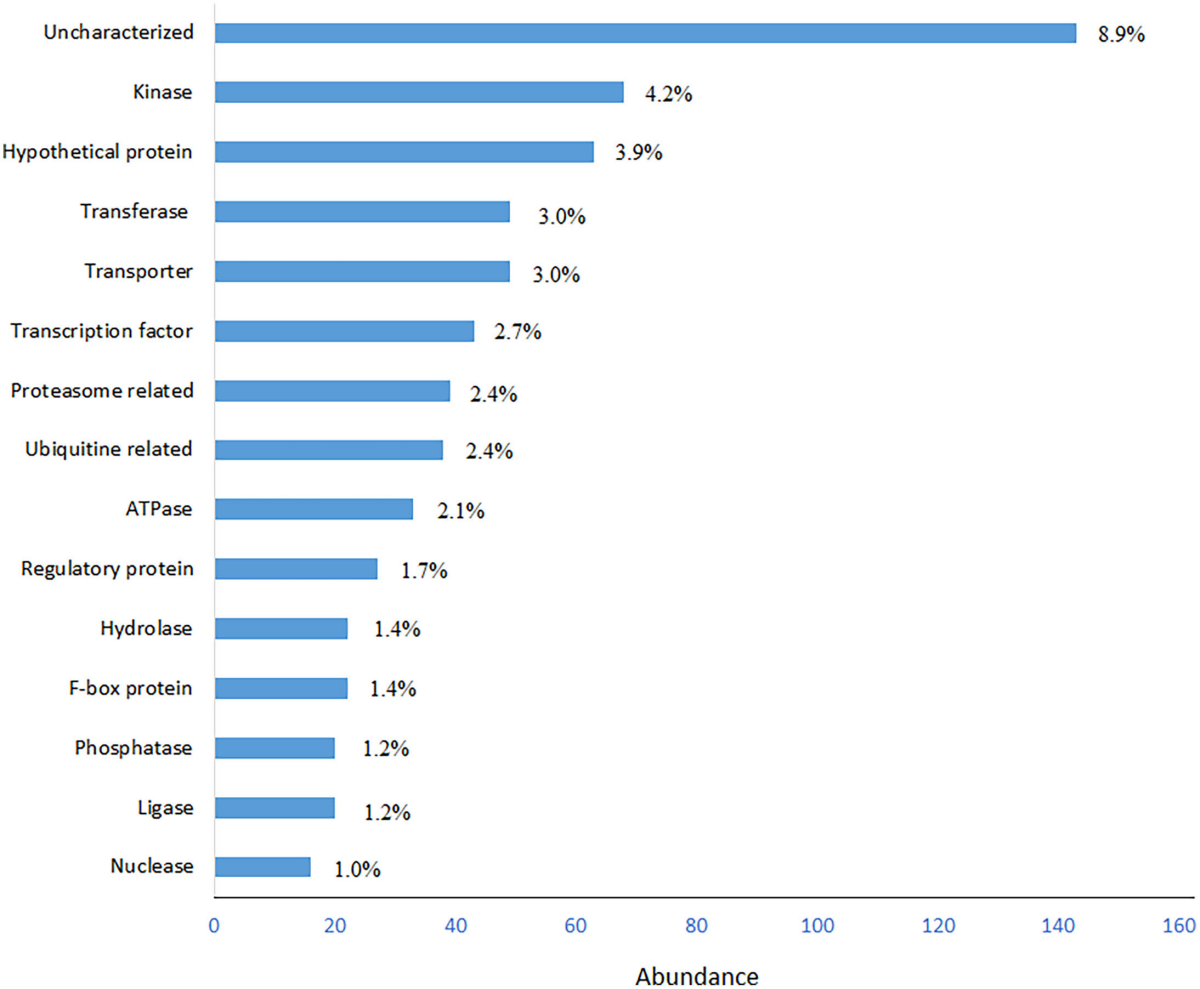
Frequency distribution of most abundant BLASTp hits of SSR-containing genes. Functional classes with ≥10 hits (in total, 652 hits) were used for drawing the graph.

652 out of 1,613 SSR-containing genes could be classified in 15 protein classes (**Figure 2**). Kinases, transferases and transportes were the most abundant protein classes in BLASTp hits. Transcription factors (TFs) also were more abundant protein classes in BLASTp hits, from which bHLH, bZIP, MYB and NAC domain containing proteins were most abundant TFs (**Supplemental Table S3**). However, the uncharacterized and hypothetical proteins were the other major portions of the analyzed SSR-containing genes that occupied 8.9% and 4.2%, respectively.

### Gene ontology and KEGG enrichment of SSR-containing genes

**Figure 3:**
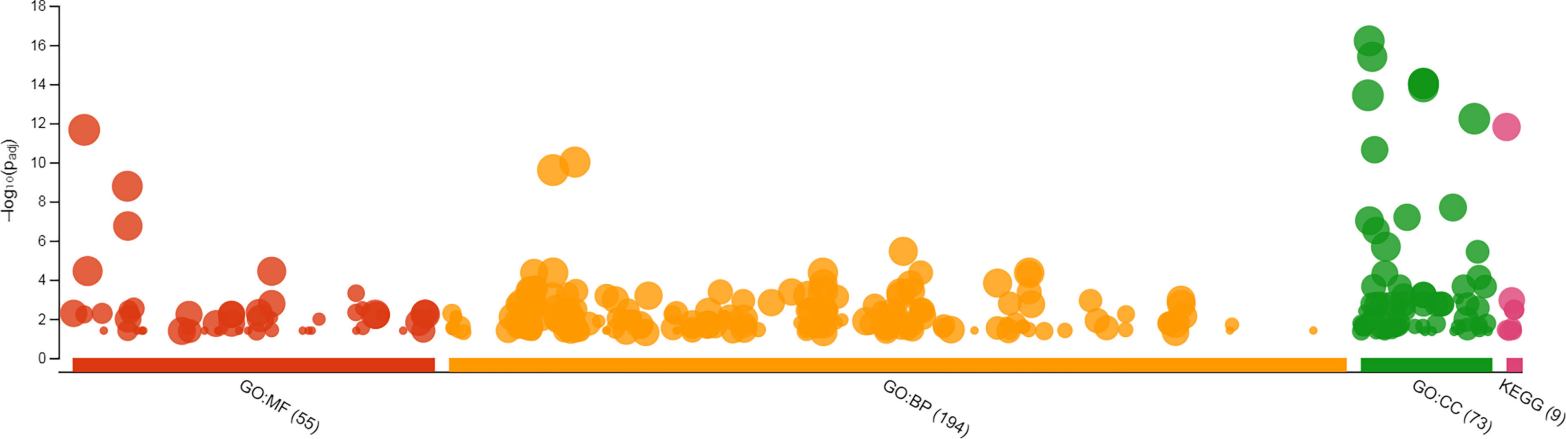
GO enrichment of safflower cDNAs containing SSR motifs by gProfiler. The magnitude of significance of hits in GO enrichment analysis is presented by –log_10_(Padj) on y axis, and the GO terms are presented on x axis. MF, molecular function; BP, biological process; CC, cellular component; KEGG, KEGG, Kyoto Encyclopedia of Genes and Genomes.

Using gProfiler tool all genes with known IDs were analyzed and only 173 genes containing mono-, di- or tri-nucleotide SSRs had hits in GO analysis and hence, were enriched for GO terms. Two genes in mononucleotide type, 145 genes in dinucleotide type, and 26 genes in trinucleotide type were enriched for GO terms. The GO-enriched genes were classified into different categories including molecular function (MF), biological process (BP) and cellular component (CC). 160, 157 and 157 genes were enriched for MF, BP and CC, respectively. In total, 322 GO terms were assigned to 173 genes: 55 GO terms for MF, 194 GO terms for BP and 73 GO terms for CC (**Figure 3**). The GO enrichment analysis and subclasses within each group with highest significant hits are presented in **Supplemental Table S4**. The results of gene ontology showed that the GO enriched genes mostly involve in biological (GO:0008150, 160 genes), cellular (GO:0009987, 132 genes) and metabolic processes (GO:0008152, 101 genes). Among molecular functions, binding (GO:0005488, 122 genes), catalytic activity (GO:0003824, 85 genes) and protein binding (GO:0005515, 78 genes), and among biological processes, organic substance metabolic process (GO:0071704, 96 genes), cellular metabolic process (GO:0044237, 94 genes), primary metabolic process (GO:0044238, 88 genes) and nitrogen compound metabolic process (GO:0006807, 80 genes) were more important subclasses; response to stimulus (GO:0050896) and biological regulation (GO:0065007) also are important biological processes. Among cellular components, cellular anatomical entity (GO:0110165, 152 genes), intracellular anatomical structure (GO:0005622, 136 genes) and organelle (GO:0043226, 124 genes) have highest importance. Note that the importance of given term(s) was deduced in view of the number of hits (genes listed for each class or subclass) in GO analysis. In KEGG enrichment analysis 67 genes were enriched in 8 different pathways, more important of which were metabolic pathways (KEGG:01100, including lipid metabolism and plant terpenoid biosynthesis), ubiquitin mediated proteolysis (KEGG:04120) and endocytosis (KEGG:04144) (**Supplemental Table S4**).

**Figure 4:**
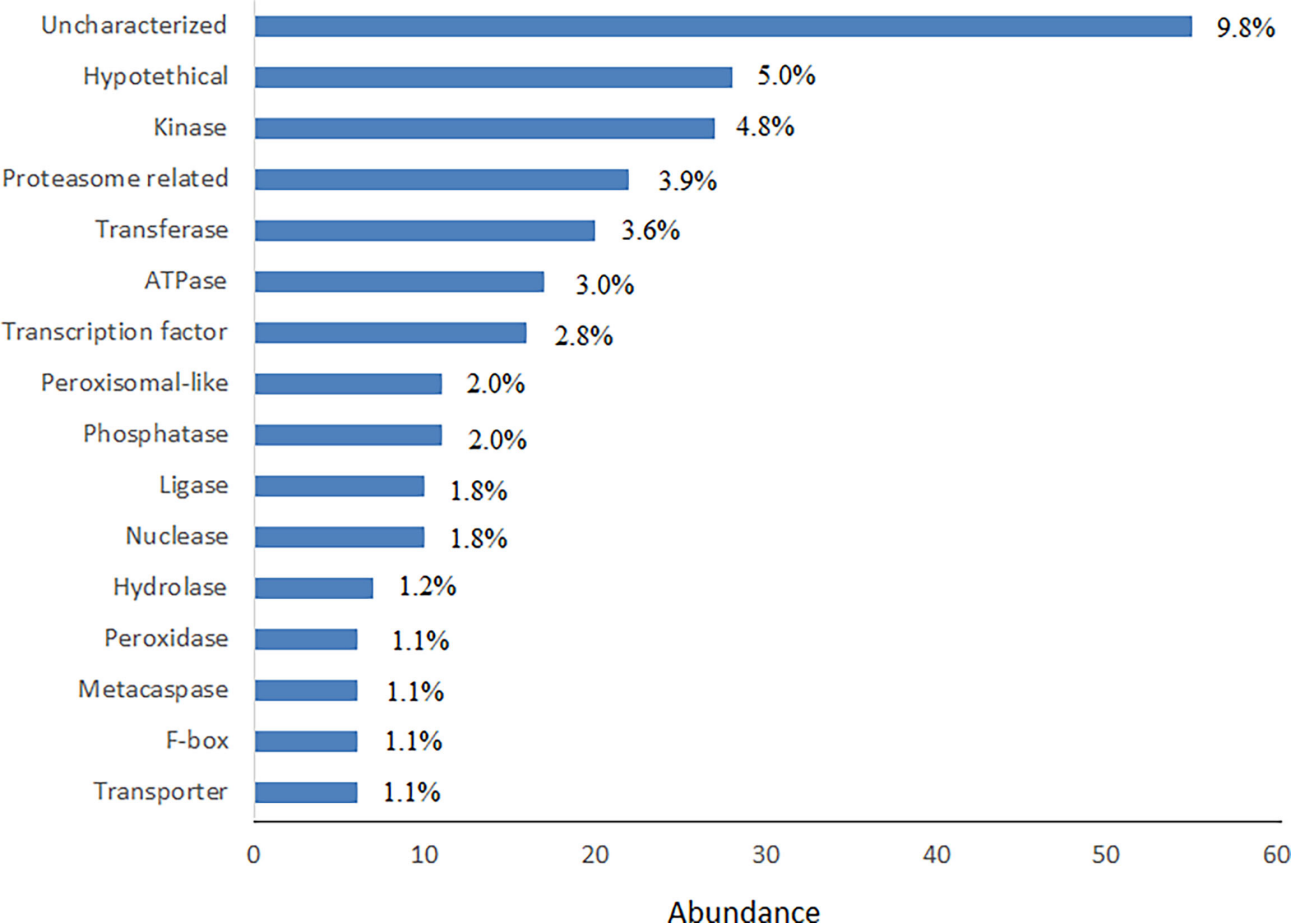
Frequency distribution of most abundant functional protein classes in trinucleotide SSR-containing genes. In total, 258 matches were used for drawing the graph.

Since trinucleotide repeats are of functional importance (Krzyzosiak and Napierala, 2011; Hao, 2018) the downstream analyses of the genes containing this type of repeats were conducted. Interestingly, all trinucleotide SSR regions which were found in 635 cDNAs, harbored perfect trinucleotide motifs (SSR length ≥20 n). 563 trinucleotide-containing genes showed significant hits in BLASTp, 258 (45.8%) of which could be grouped into 16 protein classes (**Figure 4**). Kinases, proteasome-related proteins and transferases were the most abundant protein classes which contained trinucleotide repeats in their respective coding sequences (CDS). ATPases and transcription factors (TFs) occupied next locations.

**Figure 5:**
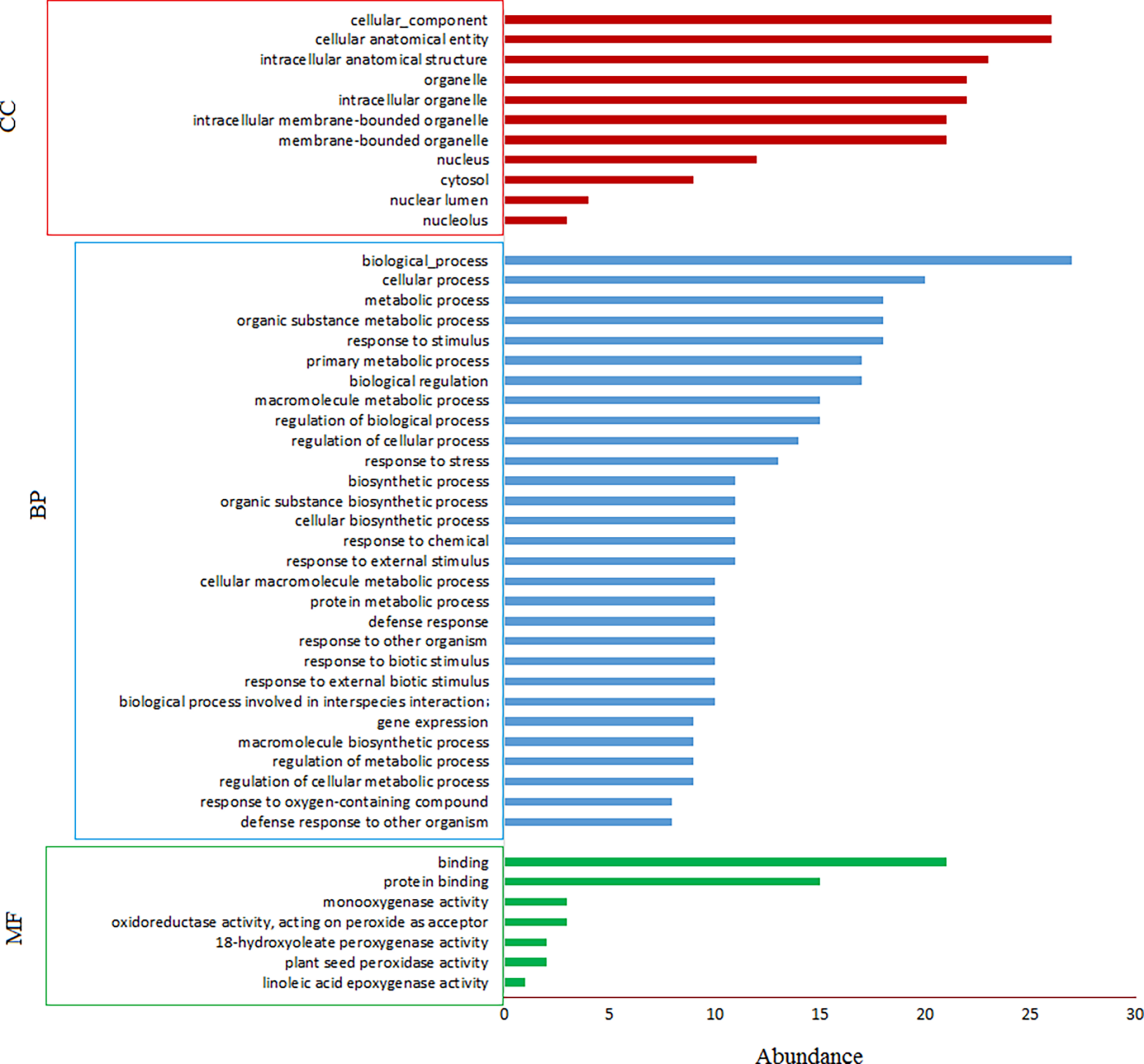
GO enrichment of safflower cDNAs containing trinucleotide SSR motifs by gProfiler. MF, molecular function; BP, biological process; CC, cellular component; KEGG, KEGG, Kyoto Encyclopedia of Genes and Genomes.

**Figure 6:**
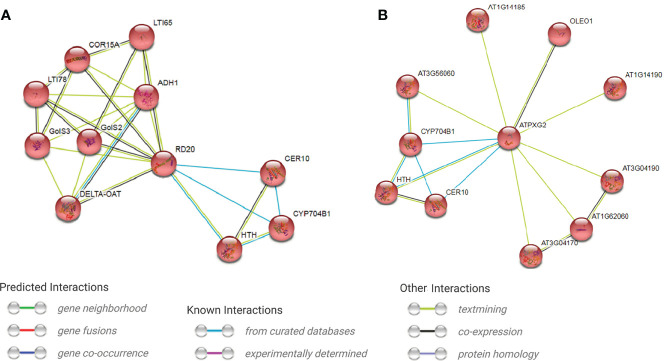
Protein networks for two key genes (containing trinucleotide SSR repeats) acting in cutin, suberine and wax biosynthesis pathway determined by MCL clustering in STRING database. **(A)** Caleosine (RD20) protein network, **(B)** Peroxygenease 2 (PXG2) protein network.

GO enrichment analysis indicated that most (77.8%) of annotated trinucleotide-containig genes have binding activity, followed by protein binding activity (55.6%) (**Figure 5**). 100% of the genes involve in biological processes, followed by cellular (74.7%) and metabolic (66.7%) processes. The considerable portion of genes also involve in organic substance metabolic process and response to stimulus (66.7%), followed by primary metabolic process and biological regulation (62.9%). Cellular anatomical entity, intracellular anatomical structure and organelle are more important cellular targets of the coded proteins of trinucleotide-containing genes. Interestingly, more than 44% of the proteins are localized to the nucleus (**Figure 5**). Based on KEGG analysis, two pathways were enriched for trinucleotide-containing genes including cutin, suberine and wax biosynthesis (KEGG:00073) and endocytosis (KEGG:04144) pathways. Because of more probable role of the first pathway in defense reactions, we would pay more attention to it. The core genes in the pathway include *Caleosin 3* (*RD20*) and *peroxygenase 2* (*PXG2*), both genes belong to Caleosin superfamily. A single cDNA clone in safflower (EL385976.1) which is mapped on chromosome 4 (at position 13.584-13.590 Mbp) carries *Caleosin 3*, and four cDNA clones (EL385118.1, EL391927.1, EL400831.1 and EL409620.1) mapped on reverse strand of chromosome 6 (at position 17.892-17.931 Mbp) carry *peroxygenase 2* (**Supplemental Table S5**). Protein interaction analysis reveals the protein networks of these two genes using STRING database (**Figures 6A, B**). As seen in the figure, both interaction networks/modules share 3 genes including CER10 (3-oxo-5-alpha-steroid 4-dehydrogenase), HTH (Glucose-methanol-choline (GMC) oxidoreductase) and CYP704B1 (a Cytochrome P450). CER10 catalyzes the last of the four reactions of the long-chain fatty acids elongation cycle. It participates in the production of very long-chain fatty acids (VLCFAs) of different chain lengths that are involved in multiple biological processes as precursor. HTH has similarity to the mandelonitrile lyase family of FAD containing oxidoreductases and is predicted to be secreted. It originally was identified as a mutation that causes floral organs to fuse together. CYP704B1 catalyzes omega-hydroxylation of long-chain fatty acids (LCFAs), implicating these molecules in sporopollenin synthesis. It is expressed in the developing anthers and is essential for pollen exine development. The complete annotations and descriptions of the proteins in these two modules presented in **Supplemental Table S6**. The analysis of *RD20* promoter region in PlantPAN (http://plantpan.itps.ncku.edu.tw/) showed that its promoter have cis-element binding sites for 3 transcription factors (viz. *ANAC019*, *ATHB-12* and *RD26*) with relatively high probability (>70%); the co-expression analysis also indicates all of the TFs are co-expressed with *RD20* (**Supplemental Table S5**). The *PXG2* promoter analysis showed that its promoter harbor many cis-element binding sites for 17 transcription factors (such as *PIL5*, *NF-AY8*, *WRKY 32* and *OPB1*) with a high probability (>90%), which also showed co-expression with it (**Supplemental Table S5**).

### Validation of SSR markers

**Figure 7:**
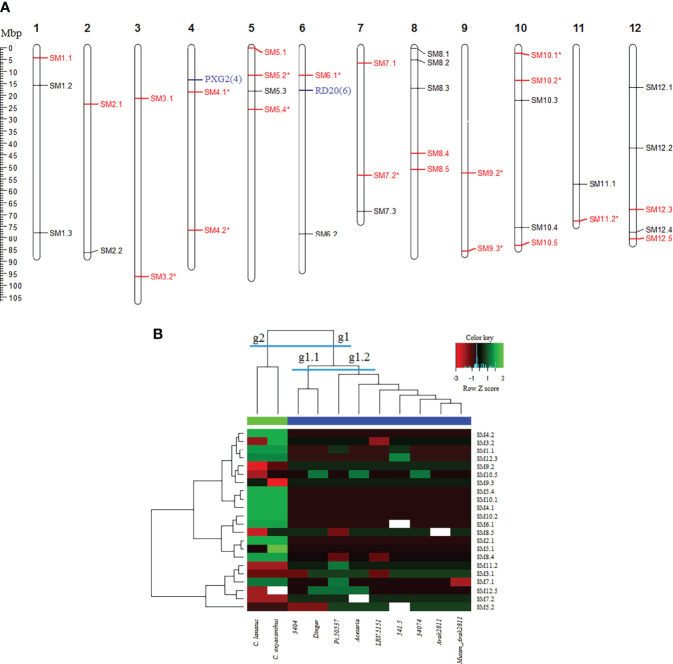
Analysis and validation of SSR markers in safflower. **(A)** Physical map positions of 38 cDNA-SSR markers in safflower genome. The map positions are in Mbp. The SSR marker names are presented on right-hand of each chromosome. Polymorphic markers are in red and the markers showing a monomorphic pattern among cultivated accessions are shown with an asterisk. Two key genes harboring trincleotide SSRs are shown in blue. **(B)** Grouping of the cultivated and wild relative accessions of safflower by 22 polymorphic cDNA-SSR markers using two-sided clustering of heat map method. Two distinct groups are defined: group g1 contains all cultivated accessions and group g2 contains 2 wild relatives (*C. lanatus* and *C. oxyacanthus*). The group g1 itself is clustered into two subgroups (subgroup g1.1 containing LRV-5151 and 34074, and subgroup g1.2 containing Arak 2811, mutant Arak 2811, Pi-50537, 3404, 541-5, Aceteria and Dinger). Green color: higher Z score values, red color: lower Z score values.

Based on cDNA sequences which contained SSR motifs, thirty eight primer pairs were designed, synthesized and used in PCR reactions for amplification of DNA of 11 safflower accessions (9 cultivated accessions of *C. carthamus* species and 2 wild relatives including *C. lanatus* and *C. oxyacanthus* species). Three primer pairs were amplified only in 4 or 5 samples (which discarded in downstream analyses), and 35 primer pairs were amplified in majority of samples, from which 22 primer pairs produced polymorphic patterns in safflower accessions. However, 12 out of these 22 SSRs were monomorphic among cultivated accessions (*e.g.* 26.3% were polymorphic among cultivated accessions). The polymorphic SSR markers had 2 to 3 alleles (**Supplemental Table S7**). All SSR markers were physically mapped on the newly published safflower genome by sequence alignment of their carrying cDNA sequences (**Figure 7A**). The safflower accessions were grouped into two distinct groups based on two-sided clustering by heatmap analysis (**Figure 7B**). All cultivated accessions placed in one group (g1) and 2 wild relatives (*C. lanatus* collected from Tehran Province and *C. oxyacanthus* collected from Golestan Province) obviously placed in a separate group (g2). The cultivated group g1 itself was clustered into two subgroups (subgroup g1.1: LRV-5151 and 34074, and subgroup g1.2: Arak 2811, mutant Arak 2811, Pi-50537, 3404, 541-5, Aceteria and Dinger).

## Discussion

In self-pollinated species like safflower there are few studies on genome structure and features, resulting to a limited number of SSR markers until now and hence, the exploitation of the marker-assisted breeding (MAB) potential is limited. For instance, 509 putative genomic SSR markers were acquired *via* next-generation sequencing (NGS), thirty of which were assessed in a diverse collection of safflower (Lee et al., 2014). In a more comprehensive NGS effort, Ambreen et al. (2015) identified 23,067 SSR regions in whole-genome sequences and validated 294 of them in a set of 23 safflower accessions. However, the identification of microsatellite sequences in genic regions of safflower, which can be done by *in silico* analysis, rarely was reported. These genic-based developed markers are functional molecular markers and cost-effective; because of their presence in conserved and semi conserved genic regions of the genome, they are expected to have high inter-specific transferability (Scott et al., 2000; Varshney et al., 2005). Genic SSR markers are the markers of choice and more robust in compare to non-genic SSRs for genetic investigation and QTL mapping (Agarwal et al., 2015). Genic SSRs, particularly cDNA-derived ones, are more reliable markers to be used in linkage and association mapping studies because they are not prone to recombination events between marker and associated trait, which decreases the occurrence of false positives in marker-assisted selection programs (Gupta et al., 2010).

Based on BLASTp results, >93% of SSR-containing cDNAs showed high similarity with the existing database sequences and only around 7% of them did not show any similarity with the existing database sequences. Similar results were reported in mulberry transcripts by Thumilan et al. (2016). 1,407 (81.5%) of cDNA-SSR sequences analyzed here had well-defined function including major protein classes such as kinase, transferase, transporter, transcription factor and regulatory protein (**Figure 2**). Thumilan et al. (2016) conducted a screening of mulberry EST sequences and identified SSR sequences in kinases, transcription factors, transporters and ribosomal proteins. Most frequent SSR-containing transcription factors in our study were bHLH, bZIP, NAC and MYB. Tranbarger et al. (2012) also identified SSRs in regulatory transcripts of oil plam (*Elaeis guineensis* Jacq.) including bZIP, zinc finger, MADS-box, and NAC-like transcription factors. Similar results reported by others in chickpea (Kujur et al., 2013), *Medicago truncatula* (Liu et al., 2015), mango (Mahato et al., 2016), jute (Satya et al., 2017) and tea (Parmar et al., 2022).

Repeat number of SSRs varied from 3 to 28, but repeat numbers of <10 were predominant in cDNA sequences. Similar results were reported for *Eucaliptus* genic SSRs by Liu et al. (2018). The effect of repeat number of SSRs in genic regions on the phenotypic performance of the genes was not studied comprehensively in plants. A preliminary study on the effect of repeat number of SSRs showed that repeat number of CT motif in rice *Waxy* gene affected amylose content of seed endosperm (Prathepha, 2003), but more recently its effect on starch properties of endosperm was reported as a suspicious issue (Li et al., 2017).

In present research the TC, AG and AC motifs were the most abundant dinucleotide motifs, and TTC, ATC and AAG were the most abundant trinucleotide motifs (**Table 1**). Liu et al. (2018) also reported that among dinucleotide motifs, TC (21.3%) and AG (20.7%) were the most frequent SSR motifs in *Eucalyptus*, which is consistent with the results of present research. In tea transcripts, AG, AT and AC motifs were most prevalent among the dinucleotide repeats, and AAG, ACC and ATC motifs were the most abundant trinucleotide repeats (Parmar et al., 2022). As the results of the present study, the motifs with thymine (T) and cytosine (C) bases showed the highest contribution in all of the identified motifs (see **Supplemental Table S2**). This issue also reported by Zhou et al. (2016) in the *Dipteronia oliver*, which could be realized among the most abundant microsatellite repeats such as dinucleotide and trinucleotide motifs.

The trinucleotide motifs were most abundant SSR type (35.7%) followed by hexa- and dinucleotide motifs (29.6 and 22.0%, respectively) in safflower SSR-containing cDNAs, which these results are similar to those found in *Larix kaempferi* by Chen et al. (2015), in which the trinucleotide motifs were the most abundant (27.3%), followed by hexa- and dinucleotide motifs, respectively. Interestingly, it was reported that the trinucleotide repeats were more frequent in coding sequences of the human genes too (Krzyzosiak and Napierala, 2011). In contrast, mono- and dinucleotide motifs were predominant in EST-SSRs obtained by RNA-seq analysis of safflower under drought stress (92.30% and 5.12%, respectively) (Sudhakar et al., 2019). This difference may be due to the use of all cDNA sequences for deriving SSR motifs in our research and the use of only drought responding transcripts for deriving SSR motifs in the mentioned work. Parmar et al. (2022) analyzed tea transcripts and reported highest abundance of di- (61.5%) and trinucleotide SSR repeats (34.8%). In coding regions, but the small indels generated by SSRs can cause frameshifts that result in truncated or elongated proteins with disrupted protein function (Lin and Kussell, 2012). Although frameshift mutations are often regarded as deleterious, under fluctuating selection (a mode of natural selection characterized by the fluctuation of the direction of selection on a given phenotype over a relatively brief period of evolutionary time) but they can be advantageous (Kashi and King, 2006). However, trinucleotide repeats do not cause frameshifts when their repeat number is changed. Thus, they are better tolerated than other SSRs in translated sequences. When these repeats are long enough, they tend to form unusual DNA structures that affect chromatin organization and DNA function (Krzyzosiak and Napierala, 2011).

Gene ontology analysis of SSR-containing genes indicated that 24.3% of genes with known gene IDs showed significant hits for GO terms. The GO enriched genes mostly involve in biological, cellular and metabolic processes; organic substance metabolic process (GO:0071704), cellular metabolic process (GO:0044237) and primary metabolic process (GO:0044238) were more important subclasses of cellular processes. These results partly were confirmed by KEGG enrichment analysis showing 41.8% of genes involve in metabolic pathways. Lulin et al. (2012) reported that most of safflower SSR-containing genes involve in various biological processes such as cellular and metabolic processes, which is consistent with the present study. In tea, involvement of most SSR-containing genes in metabolic processes and biological regulation was reported (Parmar et al., 2022). The most important molecular functions of the GO enriched genes in present study are the binding, catalytic activity and protein binding. Higher importance of the binding, enzymatic and transporter activities in EST-SSR sequences of safflower were reported earlier (Lulin et al., 2012). Similarly, the prevalence of catalytic, transcription regulation and transporter activities in SSR-containing transcripts of tea was reported (Parmar et al., 2022). GO enrichment of trinucleotide-containing genes almost followed the GO enrichment of total SSR-containing genes (see **Supplemental Table S4** and **Figure 5**). The mild difference is in subclasses of cellular processes, where response to stimulus and biological regulation have more importance in the trinucleotide-containing genes compared to the total SSR-containing genes. Kinases, proteasome related proteins, transferases and transcription factors are among the more important classes of proteins that harbor trinucleotide SSRs in the encoding genes (**Figure 4**). Length variation of trinucleotide repeats within ORFs is thought to be the fast evolutionary track to generate novel protein sequences. Some functional classes of proteins, such as protein kinases and transcription factors, took advantage of this rapid means of change more often than other protein classes (Krzyzosiak and Napierala, 2011). However, it was suggested that DNA recombination, cell cycle, replication, DNA mismatch repair, and transcription are among the cellular processes impaired by long trinucleotide repeats (Krzyzosiak and Napierala, 2011).

KEGG analysis of trinucleotide SSR-containing genes showed an important biosynthetic pathway [cutin, suberine and wax biosynthesis (KEGG:00073)] with 2 key genes including *caleosin 3* (*RD20*) and *peroxygenase 2* (*PXG2*), which are isoform and belong to Caleosin superfamily. According to GO analysis the genes are enriched for molecular functions such as calcium ion binding, peroxygenase, lipase, monooxygenase and plant seed peroxidase activities (data not shown). Caleosin/peroxygenases (*CLO/PXG*) comprise small gene families found in a wide range of plant species; these gene families have seven members in Arabidopsis genome and five members in rice genome (Khalil et al., 2014), and are present in the vast majority of viridiplantae taxa (Rahman et al., 2018). In plants, structural proteins, particularly caleosins and oleosins affect the biological functions of seed oil bodies (OBs) (Shao et al., 2019). Protein interaction analysis to find probable existing networks consisted of the two protein RD20 and PXG2 was resulted in identification of two protein interaction networks/modules (**Figure 6A, B**), which are interconnected by 3 common members including CER10, HTH and CYP704B1 (CHC submodule). It seems that each of RD20/or PXG2 plays a vital role in its module and acts as an intermediary factor between the two sides of same network; one side consisted of a three-protein submodule CHC, and the second side consisted of a larger set of proteins which presumably along with RD20/or PXG2 determines the specified action of each network. These family members have roles in in seed germination (Poxleitner et al., 2006), lipid droplet storage and abiotic stress tolerance (Khalil et al., 2014).

Interestingly, the proteins RD20 and PXG2 are targeted to lipid bodies. On the basis of annotations and descriptions of the proteins depicted in STRING database (**Supplemental Table S6**), the biological role of the two modules can be understood. The genes in both modules mainly cooperate to manipulate/elongate seed oil bodies, particularly under environmental stresses. Biological function of these two modules apparently is endowment of tolerance to abiotic stresses such as cold, drought and salt. Rahman et al. (2018) suggested that algal CLO/PXGs play a roles in lipid packaging and stress responses, and Khalil et al. (2014) reported the differential expression of the caleosin gene family in wheat during different developmental stages and also in response to environmental stresses. Three transcription factors (*ANAC019*, *ATHB-12* and *RD26*) are co-expressed with *RD20* and bind to its promoter *via* sis-element binding sites (**Supplemental Table S5**). As reported, these TFs play a vital role in tolerance to abiotic stresses such as drought and salt (Ré et al., 2014; Hur et al., 2015; Ye et al., 2017; Jiang et al., 2019; Duan et al., 2019; Sukiran et al., 2019). Seventeen transcription factors (such as PIL5, NF-AY8, WRKY 32 and OPB1) are co-expressed with *PXG2* and bind to its promoter region (**Supplemental Table S5**). Some of these TFs have important roles in gibberellin response and germination (Oh et al., 2004; Oh et al., 2007) and floral transition and flowering (Wu et al., 2018; Chandler and Werr, 2020), but most of them improve tolerance to abiotic stresses such as drought (Singh and Laxmi, 2015; Li et al., 2016; Zhang et al., 2017; Huo et al., 2021), salt (Li et al., 2016) and photooxidative stress (Hackenberg et al., 2012) or combined biotic and abiotic stresses (Bai et al., 2018).

Most of the assayed SSR primer pairs designed based on flanking regions of SSR motifs within cDNA sequences, were robust (35 out of 38, >90%) and successfully amplified the DNA of the studied accessions; more than 57% of these genic SSRs showed polymorphism among safflower accessions. In the case of SSR markers developed based on whole genome sequencing, lower polymorphism rate (31.6%) was reported (Ambreen et al., 2015). Lower polymorphism rates in EST-SSRs and transcript-SSRs also was reported by others (Kujur et al., 2013; Mahato et al., 2016; Satya et al., 2017; Parmar et al., 2022). However, if the two wild relatives are discarded and just the cultivated accessions considered, the polymorphism rate of the SSR marker set is ~26%, which is near the rates reported by others. The polymorphic SSRs could clearly differentiate cultivated accessions from wild relatives which is consistent with classic morphological taxonomy (**Figure 7B**). This show the suitability of cDNA-SSR markers for purposes of variety identification and plant molecular systematics (Sharifi Tehrani et al., 2009; Kordrostami et al., 2016; Liu et al., 2018; Zhang et al., 2020).

## Conclusion

Occurrence of SSR repeats in biologically-important classes of proteins such as kinases and transcription factors, and variability of SSR repeat copies, endow the cell and whole organism the flexibility of facing with continuously changing endogenous and exogenous environment. The fact indicates a structure-based evolution mechanism of the genome, which acts as an up-to-dating tool for the cell to face with surrounding environment. This conclusion is realized in GO terms such as involvement of most SSR-containing genes, particularly trinucleotide-containing ones, in biological, cellular and metabolic processes, and especially in response to stimulus, response to stress, interaction to other organisms and defense responses. Present study also shows that cDNA-derived SSRs have high robustness and detect a relatively high polymorphism rate among safflower accessions and hence, are suitable for evaluation of genetic diversity, linkage and association mapping studies and genome-based breeding programmes.

## Data availability statement

The datasets presented in this study can be found in online repositories. The names of the repository/repositories and accession number(s) can be found in the article/**Supplementary Material**.

## Author contributions

AhA conducted the research and prepared the draft of manuscript. AsA conceptualized and supervised the research and edited the final manuscript. All authors contributed to the article and approved the submitted version.

## Conflict of interest

The authors declare that the research was conducted in the absence of any commercial or financial relationships that could be construed as a potential conflict of interest.

## Publisher’s note

All claims expressed in this article are solely those of the authors and do not necessarily represent those of their affiliated organizations, or those of the publisher, the editors and the reviewers. Any product that may be evaluated in this article, or claim that may be made by its manufacturer, is not guaranteed or endorsed by the publisher.

## Abbreviations

AFLP, amplified fragment length polymorphism; BP, biological process; BLAST, basic local alignment search tool; CC, cellular component; EST, expressed sequence tag; GO, gene ontology; ISSR, inter simple sequence repeat; KEGG, Kyoto encyclopedia of genes and genomes; LA, linoleic acid; MF, molecular function; OA, oleic acid; SSR, simple sequence repeat; QTL, quantitative trait loci; RAPD, random amplified polymorphic DNA; SCoT, start codon targeted; SRAP, sequence-related amplified polymorphism.
