# Supplementary material for: Occurrence of simple sequence repeats in cDNA sequences of safflower (Carthamus tinctorius) reveals the importance of SSR-containing genes for cell biology and dynamic response to environmental cues
Source: Front Plant Sci. 2022 Nov 17;13:991107. doi: 10.3389/fpls.2022.991107 (PMC9714374; doi:10.3389/fpls.2022.991107)
Supplement: Supplementary file 1 [file DataSheet_1.docx]

**Supplemental File S1.** R packages and scripts used in the study.

**#1:** A sample of R scripts used for identification of SSR motifs in cDNA sequences of safflower.

#1-1 using matchPDict() function

setwd("./working_directory")

library(Biostrings)

cDNA<- readDNAStringSet("./cDNA_sequences.fasta")

dict <- readDNAStringSet("./sample_dinucletide_motifs.txt")

pdict <- PDict(dict, tb.end=10)

ssr2n <- matchPDict(pdict, DNAString(toString(cDNA)), max.mismatch=0)

#Note: a sample of dinucletide motifs with minimum length of 18 bases (which is retained in the file “sample_dinucletide_motifs.txt”) is as following:

>dinucl1

ATATATATATATATATAT

> dinucl2

ACACACACACACACACAC

> dinucl3

AGAGAGAGAGAGAGAGAG

> dinucl4

TCTCTCTCTCTCTCTCTC

> dinucl5

TGTGTGTGTGTGTGTGTG

> dinucl6

GCGCGCGCGCGCGCGCGC

#2: R scripts used for physical mapping of SSR sequences onto safflower genome

#2-1 using matchProbePair() function

setwd("./working_directory")

library(Biostrings)

subject <- readDNAStringSet(./safflower_genome.fasta”)

Fprobe<-"XXXXXXXXXXXXXXXXXX"

Rprobe<-"XXXXXXXXXXXXXXXXXX"

match2primers <- matchProbePair(Fprobe, Rprobe, DNAString(toString(subject[1])))

# note: subject[1] is chromosome number 1

#2-2 using matchLRPatterns () function

setwd("./working_directory")

library(Biostrings)

match2primers<-matchLRPatterns(Lpattern, Rpattern, max.gaplength=10000, DNAString(toString(subject[1])), max.Lmismatch=3, max.Rmismatch=3, with.Lindels=FALSE, with.Rindels=FALSE,Lfixed=TRUE, Rfixed=TRUE)
